# Post-MI Remodeling Mechanics of Left Ventricle: Microstructure-Informed Models, Identifiability, and Uncertainty for Patient-Specific Prediction

**DOI:** 10.3390/bioengineering13090991

**Published:** 2026-08-27

**Authors:** Thanyani Pandelani, Fulufhelo Nemavhola

**Affiliations:** 1 Department of Mechanical, Bioresources and Biomedical Engineering, School of Engineering, College of Science and Engineering Technology, University of South Africa, Unisa Science Campus, Florida Park, Johannesburg 1709, South Africa; 2 Department of Mechanical Engineering, Faculty of Engineering and the Built Environment, Durban University of Technology, Durban 4001, South Africa

**Keywords:** myocardial infarction, ventricular remodeling, growth and remodeling, constitutive modeling, fibrosis, collagen, border zone, cardiac magnetic resonance

## Abstract

Background: Myocardial infarction (MI) causes spatially heterogeneous loss of contractility and progressive extracellular matrix remodeling, altering left ventricular mechanics from the acute phase through chronic remodeling. This review integrates current understanding of infarct, border-zone, and remote-myocardial microstructure with organ-scale mechanics and patient-specific computational modeling. Methods: A narrative review and perspective were conducted using the literature identified through PubMed/MEDLINE, Scopus, and Web of Science, supplemented by targeted searches of IEEE Xplore and Google Scholar. Experimental, imaging, computational, and translational studies were synthesised, with emphasis on post-MI constitutive behaviour, finite-element and growth-and-remodeling models, imaging-informed personalization, inverse parameter estimation, identifiability, model calibration, verification and validation, and uncertainty quantification. No quantitative synthesis was performed because of substantial heterogeneity in study populations, imaging modalities, constitutive formulations, boundary conditions, calibration procedures, and reported outcomes. Results: Contemporary post-MI models can reproduce ventricular volumes, regional strain patterns, and selected haemodynamic measures, while enabling counterfactual simulations of infarct stiffness, border-zone contractility, and loading interventions. However, clinically credible prediction remains constrained by limited in vivo observability of regional tissue properties, poor parameter identifiability, confounding between material properties and loading conditions, and incomplete treatment of measurement, parameter, and model-form uncertainty. Conclusions: The novelty of this review lies in framing post-MI patient-specific modeling as an identifiability- and uncertainty-limited inverse problem rather than solely as a model-fitting exercise. It proposes that translation toward decision-grade prediction requires parsimonious models aligned with a defined clinical context of use, constrained by microstructure-informed priors, multimodal pressure–volume–strain data, longitudinal validation, and routine reporting of parameter identifiability and predictive uncertainty.

## 1. Introduction: Mechanics as a Unifying Language for Post-MI Remodeling

The mechanical state of the left ventricle is not merely a consequence of myocardial infarction; it is a driver of the subsequent biological response. The abrupt cessation of active tension generation in the infarcted region changes regional stress and strain within a single cardiac cycle [1,2,3]. These altered mechanical fields then persist across healing and chronic remodeling, shaping fibroblast activation, extracellular matrix deposition and alignment, myocyte hypertrophy in the remote myocardium, and the evolution of chamber geometry [2,4,5,6,7,8,9,10,11,12,13,14,15,16,17,18]. Clinically, this appears as a spectrum of outcomes. Some patients show limited chamber dilation and regain functional reserve, whereas others develop progressive enlargement, elevated filling pressures, functional mitral regurgitation, and arrhythmia risk, ultimately meeting criteria for heart failure with reduced ejection fraction [1,7,18,19,20,21,22]. Classic clinical frameworks emphasise infarct size, reperfusion timing, and neurohormonal activation [1,19], but these determinants operate through mechanical mediation: the distribution of regional strain, the evolution of passive stiffness, and the remodeling of geometry that changes the heart’s loading problem [1,2,4,19,23,24,25,26,27,28,29,30,31,32,33,34,35,36,37,38,39,40,41,42,43].

A mechanical view is attractive because it integrates multiple biological scales. At the microscale, myocyte sarcomeres generate force and sense stretch, while collagen fibrils and cross-links set stiffness at higher strains; the organization of fibers and laminar sheets then creates anisotropy [36,37]. At the mesoscale, the infarct border zone contains viable but impaired myocytes embedded in heterogeneous fibrosis, producing strong mechanical gradients [7,44,45,46,47,48,49,50]. At the organ scale, ventricular shape, wall thickness, and boundary constraints determine how pressure translates to stress and how regional dysfunction influences global pump performance [1,5,6,19,23,24,25,26,29,30,31,32,35,36,38,39,51,52]. At the system scale, afterload and preload are set by vascular properties, volume status, and valvular competence, feeding back to ventricular wall stress and remodeling trajectories [1,5,6,10,13,19,23,24,25,26,29,30,31,32,35,36,38,39,49,50,51,52].

Computational modelling has increasingly been used to connect these scales. Ventricular finite-element models can incorporate patient-specific geometry, rule-based or measured fibre architecture, constitutive descriptions of passive and active tissue behaviour, and boundary conditions that approximate pressure loading and basal constraints [31,35]. Growth and remodelling formulations extend this framework beyond instantaneous mechanics to time-evolving structural adaptation, including collagen turnover, fibre reorientation, myocyte hypertrophy, and changes in residual stress [53,54]. Alternative computational approaches, including meshless methods such as Smoothed Particle Hydrodynamics and Element-Free Galerkin formulations, isogeometric analysis, fluid–structure interaction models, and hybrid neural network–finite-element frameworks, are also increasingly relevant to cardiac biomechanics. These approaches may offer particular advantages for representing large deformations, complex ventricular geometries, coupled ventricular haemodynamics and valve dynamics, and computationally efficient surrogate prediction. However, the present review focuses primarily on finite-element-based solid-mechanics and growth-and-remodelling models because these approaches currently provide the most established framework for patient-specific parameter estimation, constitutive modelling, and inverse analysis in post-MI ventricular mechanics.

These models are compelling because they allow counterfactual questions to be explored: how would remodelling change if infarct stiffness were increased early, if contractility were improved in border-zone myocardium, or if afterload were reduced in a particular patient? Such questions motivate the translational promise of patient-specific prediction for risk stratification and therapy planning [1,5,6,10,13,19,23,24,25,26,29,30,31,32,35,36,38,39,49,50,51,52]. However, the post-MI modeling field sits at a familiar translational inflection. Demonstrations of fit are common, but clinically credible prediction is rare. This gap is not primarily due to insufficient computational power, but to observability and identifiability [55,56,57,58,59,60,61,62,63,64]. In this context, identifiability refers to whether the available clinical or experimental measurements contain sufficient information to estimate the model parameters uniquely and reliably. When a model is poorly identifiable, different combinations of parameters may reproduce the same observed data, resulting in uncertain parameter estimates and potentially non-unique predictions. Clinical imaging provides geometry and motion; late gadolinium enhancement identifies scar distribution; and strain estimation provides regional deformation patterns. Yet these data do not uniquely identify material parameters and active function, especially when ventricular pressure is uncertain or unmeasured [55,56,57,58,59,60,61,62,63,64]. Consequently, multiple parameter combinations can reproduce observed kinematics with similar error [56,57,61,64]. A model that fits may not be a model that is correct, and a model that is correct in one context may not generalize to another patient or to intervention scenarios [56,60,61]. The challenge is to design model and measurement combinations that support inference with transparent uncertainty, aligned to specific decision tasks [56,57,59,60,61].

The central challenge in post-MI mechanics is not only building physiologically rich models, but designing model–measurement combinations that support inference with transparent uncertainty and are aligned with specific clinical decision tasks. In this review, “decision-grade prediction” refers to a model output that has been sufficiently verified, independently validated, and uncertainty-quantified to support a clearly defined clinical decision. This requires agreement with clinically relevant endpoints in representative patient cohorts, transparent reporting of measurement, parameter, and model-form uncertainty, and evidence that the model provides reliable information beyond established clinical predictors. This review therefore synthesises post-MI remodeling mechanics from microstructure to patient-specific prediction, with emphasis on four topics. First, we summarise microstructure-informed constitutive modeling for infarct, border zone, and remote myocardium, highlighting how collagen architecture, cross-linking, and fiber dispersion govern macroscopic stiffness and anisotropy. Second, we analyse growth-and-remodeling laws for healing and long-horizon remodeling, distinguishing components that are well supported from those that remain conjectural. Third, we describe practical personalization pipelines integrating Cardiovascular Magnetic Resonance (CMR), echocardiography, Computed Topology (CT), and emerging approaches such as diffusion Magnetic Resonance Imaging (MRI) and elastography, explicitly treating measurement uncertainty rather than assuming fixed inputs. Fourth, we position parameter identifiability and uncertainty quantification as central requirements for translation. We conclude with a measurement agenda intended to move the field toward clinically credible prediction—paired pressure–volume–strain datasets, calibrated imaging-to-mechanics biomarkers, longitudinal follow-up, and validation against endpoints that matter to clinicians [4,5,6,29,30,31,32].

**Figure 1:**
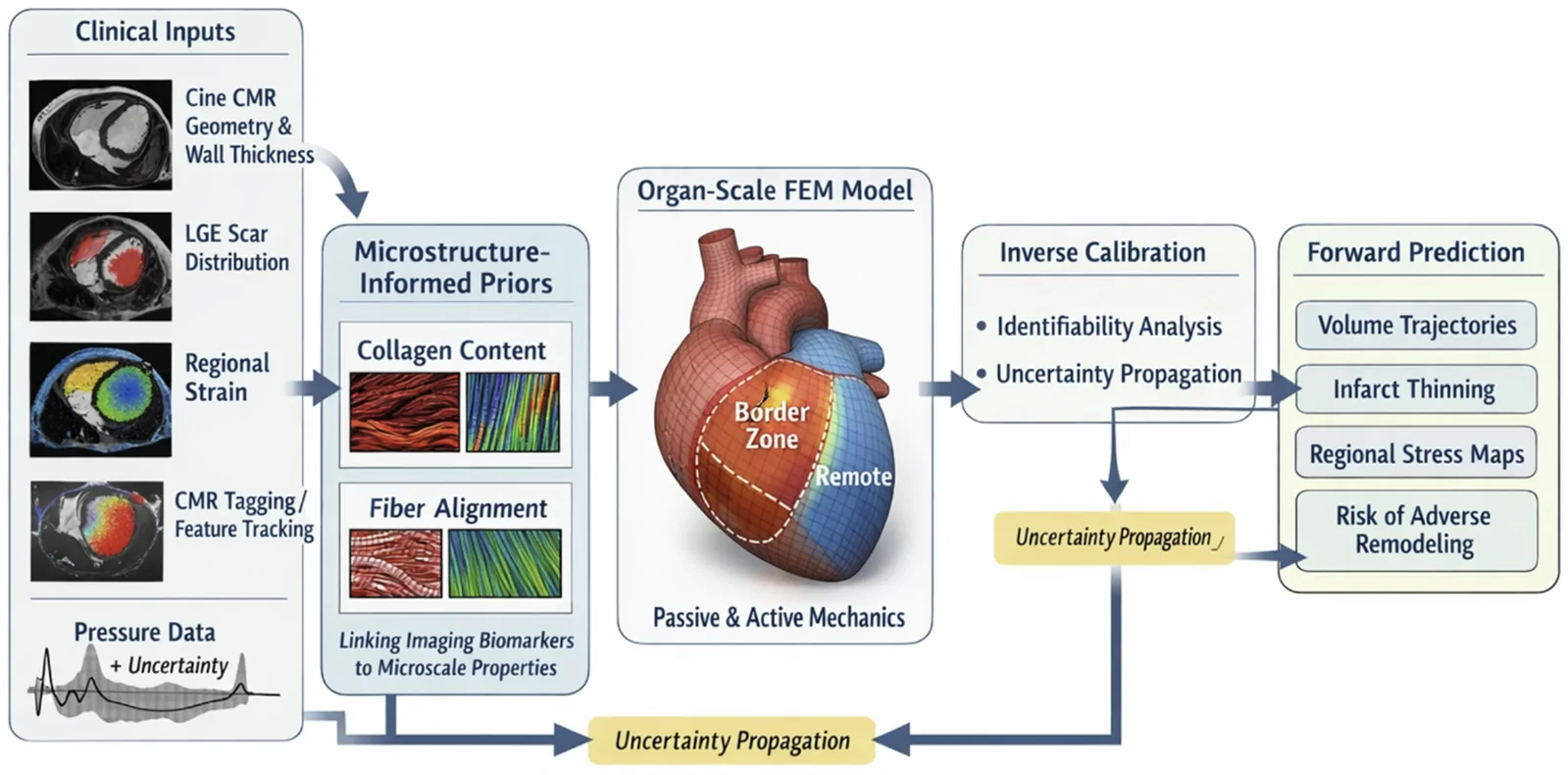
Schematic of a multiscale, imaging-informed personalization pipeline for post-MI mechanics. Clinical inputs include cine CMR-derived geometry, wall thickness, LGE-defined scar extent, regional strain, and uncertain pressure data. These measurements inform microstructure-based priors that constrain a finite-element model comprising infarct, border-zone, and remote myocardium. Patient-specific parameters are estimated using inverse calibration with identifiability analysis and uncertainty quantification, producing parameter distributions rather than single best-fit values. The calibrated model predicts remodeling outcomes, including ventricular volume, infarct thinning, regional stress and strain, and adverse-remodeling risk, while tracking how uncertainty propagates through the workflow. Abbreviations: MI, myocardial infarction; CMR, cardiac magnetic resonance; LGE, late gadolinium enhancement. Accordingly, the central argument of this review is that adding microstructural detail does not, by itself, produce clinically credible prediction. Microstructure-informed models can support patient-specific decision-making only when the parameters they introduce are identifiable from available clinical measurements and when measurement, parameter, and model-form uncertainty are explicitly quantified and propagated to clinically relevant outcomes.

As summarized in Figure 1, a decision-grade post-MI mechanics workflow is best framed as an end-to-end personalization pipeline rather than a single calibration step: routine clinical measurements (cine CMR geometry and wall thickness, late gadolinium enhancement (LGE) scar distribution, regional strain from tagging/feature tracking, and hemodynamic inputs with explicit uncertainty) are first translated into microstructure-informed priors that constrain regional material behavior across infarct core, border zone, and remote myocardium. These priors then inform an organ-scale finite-element model that is calibrated using inverse methods augmented with identifiability checks and uncertainty propagation, so that parameter estimates are interpreted as probability distributions and not point values. The same pipeline supports forward predictions that map directly onto remodeling-relevant outcomes, such as volume trajectories, infarct thinning, regional stress maps, and adverse-remodeling risk, while making uncertainty entry points and their downstream effects transparent for clinical interpretation.

## 2. Literature Search and Scope

This manuscript presents a narrative review and perspective on the mechanics of post-myocardial infarction ventricular remodeling and the development of patient-specific computational models. The literature search was conducted to identify representative, influential, and methodologically relevant publications rather than to produce an exhaustive systematic review.

The relevant literature was identified through searches of PubMed/MEDLINE, Scopus, and Web of Science. These searches were supplemented by targeted searches in IEEE Xplore and Google Scholar to capture studies relating to computational biomechanics, numerical methods, parameter estimation, and inverse-problem methodologies that may not be comprehensively indexed in biomedical databases.

The search strategy used combinations of controlled vocabulary and relevant keywords, including myocardial infarction, ventricular remodeling, finite element modelling, patient-specific modelling, constitutive modelling, growth and remodeling, mechanobiology, inverse modelling, parameter identifiability, Bayesian calibration, uncertainty quantification, verification and validation, cardiac magnetic resonance imaging, MRI tagging, Displacement Encoding with Stimulated Echoes, DENSE, feature tracking, late gadolinium enhancement, LGE, T1 mapping, and extracellular volume or ECV. Search terms were adapted to the indexing conventions and functionality of each database.

The selection of literature was guided by the scope and objectives of the review. Priority was given to: (i) seminal mechanistic and experimental studies describing the mechanical evolution of the myocardium following myocardial infarction; (ii) clinically grounded computational studies incorporating human imaging, anatomical, or haemodynamic data; and (iii) recent methodological studies addressing parameter estimation, identifiability, model calibration, uncertainty quantification, and model verification and validation.

“Seminal” studies are defined as early or highly influential investigations that introduced, established, or substantially advanced a major experimental, constitutive, computational, or mechanobiological concept relevant to post-infarction ventricular remodeling. “Clinically anchored” studies are defined as computational investigations that incorporated patient-derived or human clinical data, such as cardiac magnetic resonance imaging, ventricular geometry, scar characterization, myocardial motion, pressure measurements, haemodynamic information, or other subject-specific physiological data.

Studies concerned exclusively with cardiac electrophysiology, without a substantive mechanical-modelling component, were generally outside the scope of the review. Studies focused primarily on congenital cardiac remodeling or unrelated cardiac pathologies were also not considered unless they provided transferable methodological insights relevant to post-infarction mechanics or patient-specific modelling. Publications lacking sufficient methodological detail to evaluate the modelling assumptions, parameter-inference approach, or interpretation of results were given limited emphasis.

The reference lists of key publications and relevant review articles were also examined to identify additional influential studies. Because this article is a narrative review and perspective, the literature was synthesized thematically and critically rather than through formal systematic screening, risk-of-bias assessment, or PRISMA-based study selection. The search and selection process was therefore intended to provide a transparent and balanced overview of the major concepts, methods, limitations, and emerging research directions within the field.

**Figure 2:**
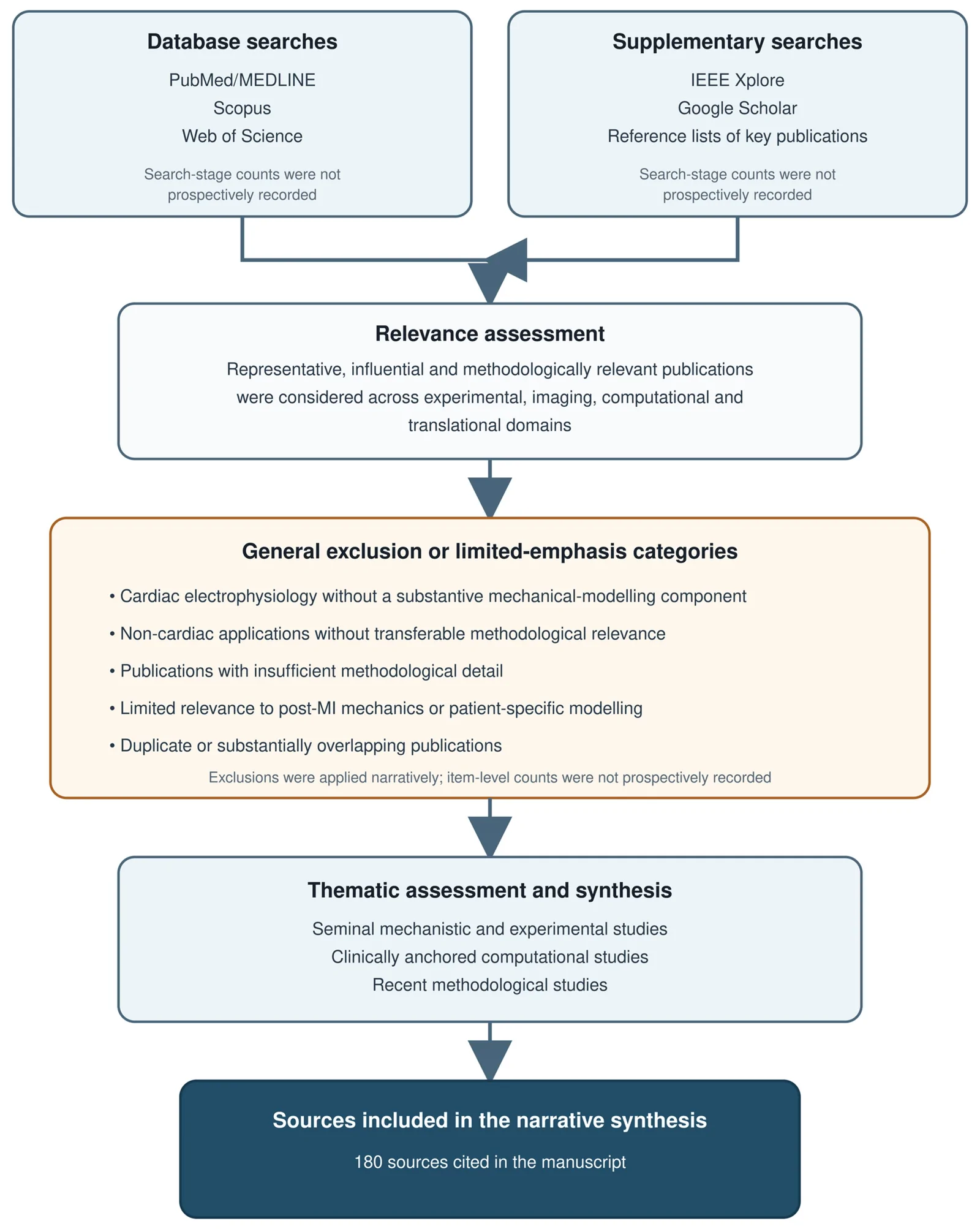
Approximate literature search and selection process. The values shown were reconstructed retrospectively from the authors’ search records, working literature libraries, reference-management records, and final bibliography. They provide an approximate representation of the scope and logic of literature selection for this narrative review and should not be interpreted as a PRISMA-compliant record of systematic screening.

A simplified overview of the literature search and selection process is provided in Figure 2. Because the review was conceived as a narrative review, numerical search and screening records were not prospectively maintained. The values presented in the figure were therefore reconstructed retrospectively from the authors’ search records, working literature libraries, reference-management records, and the final bibliography. They should be interpreted as approximate indicators of the scope and logic of the literature selection rather than as a PRISMA-compliant record of systematic screening.

## 3. The Remodeling Timeline: Linking Biological Phases to Mechanical Transitions

The post-MI remodeling process is often described using biological phases, but each phase has a characteristic mechanical signature that should guide modeling choices and data collection [1,4,5,19]. The acute phase spans hours to days [1,2,4]. Within minutes, myocyte death eliminates active tension generation in the ischemic region, producing systolic bulging and altered load redistribution [1,2,3]. Within hours, edema, hemorrhage, and inflammatory infiltration change the tissue’s effective stiffness and damping, while early degradation of matrix components reduces structural integrity [2,65,66,67]. Mechanical vulnerability is high during this period: elevated stress and strain in the infarct may drive infarct expansion, wall thinning, and, in severe cases, rupture [3,19,68,69]. The mechanical environment is also highly time-dependent, because the composition and fluid content of the tissue can change rapidly [2,65,70,71]. Models focused on the acute phase must therefore consider not only passive stiffness but also viscoelastic or poroelastic effects and evolving boundary conditions as hemodynamics shift [7,36,37,54,72,73].

The proliferative or granulation phase typically spans days to weeks [2,4,5,8]. In this phase, fibroblasts differentiate into myofibroblasts and deposit collagen-rich extracellular matrix, providing mechanical reinforcement [9,10,11,74]. Collagen deposition is not uniform; it evolves spatially with gradients from the infarct core to the border zone and through the wall thickness, and it evolves temporally in content, alignment, and cross-link maturity [2,8,34,74]. Infarct stiffness tends to increase during this period, while active contraction remains absent in the infarct core [2,12,13,34]. The border zone experiences complex mechanics: it contains viable myocytes and can contract, but contraction is impaired and often asynchronous, and fibrosis introduces anisotropy and stiffness heterogeneity [8,44,45,46]. Mechanical gradients are steep, making this region a likely driver of continued remodeling and an important substrate for arrhythmia [8,44,75,76].

The chronic maturation phase extends from weeks to months and, in some patients, years [1,2,8,19]. Scar collagen becomes more organized, cross-link density increases, and the scar’s constitutive response may become stiffer and less dissipative [1,2,8,19,34]. At the same time, remote myocardium adapts to altered loading through hypertrophy and often through diffuse interstitial fibrosis [14,15,16,19]. These changes increase passive stiffness and can impair diastolic filling [1,15,16,17]. Ventricular geometry may continue to dilate, and the valve apparatus may remodel secondarily, producing functional mitral regurgitation that further increases volume load [1,18,20,21]. Thus, chronic remodeling is not simply a stable scar; it is an evolving global mechanical problem with multiple coupled components [1,8,19,22].

Mechanically, the remodeling process can be understood as two coupled feedback loops [1,8,19,22]. The first is local feedback: infarct compliance influences infarct stretch; infarct stretch influences collagen deposition and alignment; collagen deposition and cross-linking influence infarct stiffness [2,3,8,77]. If early compliance is high and stiffening is delayed, infarct expansion and thinning can proceed, setting the stage for adverse remodeling [2,3,19,68]. If early stiffening occurs and expansion is limited, global remodeling may be attenuated [8,34,77,78]. The second is global feedback: altered regional stiffness and contractility reshape overall wall stress distribution, promoting hypertrophy and fibrosis in remote myocardium; these changes alter chamber stiffness and geometry, changing the wall stress distribution further [14,15,19,22]. Therapies such as Angiotensin-Converting Enzyme (ACE) inhibitors, beta-blockers, and mineralocorticoid receptor antagonists reduce neurohormonal drive and alter loading conditions, effectively modifying the mechanical inputs into these feedback loops [19,79,80].

From a modeling perspective, these phases imply that different constitutive assumptions and remodeling laws may be appropriate at different times [2,22,36,37,54,81]. Acute models may need to represent edema-related compliance changes and nonlinear viscoelastic behaviour [2,54,65,73]. Subacute models must represent rapid collagen deposition and alignment changes, requiring time-dependent material parameters or mixture-based formulations [31,34,74,77,82]. Chronic models must represent stable but heterogeneous scar properties, evolving remote fibrosis, and changes in wall thickness due to hypertrophy [8,15,16,22]. If a model is used for patient-specific prediction, its context of use must specify the time window and clinical decision, because the required fidelity and the relevant uncertainties differ across phases [83,84,85,86,87].

Table 1 summarises the key point for model design: post-MI mechanics are strongly phase- and region-dependent. Rather than restating the table, we use it to motivate time-window–specific modeling choices: acute representations should accommodate edema-related compliance changes and early expansion risk; proliferative-phase models should capture rapid collagen deposition/alignment and evolving heterogeneity; and chronic-phase models should represent a stabilized but spatially heterogeneous scar alongside progressive remote remodeling. These phase-appropriate assumptions help align model complexity with the dominant mechanisms and uncertainties relevant to the intended clinical context of use.

## 4. Microstructure-to-Mechanics in Infarct, Border Zone, and Remote Myocardium

Healthy myocardium is mechanically anisotropic because its load-bearing architecture is anisotropic. Myocytes are aligned into fiber bundles that rotate transmurally, and laminar sheets provide preferred planes of shear. The extracellular matrix forms a collagen network that couples cells, transfers force, and limits deformation at higher strains. Passive mechanical response is therefore nonlinear and direction-dependent: at low strains, myocyte and cytoskeletal components and titin contribute substantially, whereas at higher strains collagen recruitment dominates. Viscoelasticity arises from fluid–solid interactions, cross-bridge cycling history, and intrinsic matrix behavior. Constitutive models that aim to represent healthy myocardium often encode this architecture using one or more preferred directions and nonlinear stiffening terms [36,37]. Post-MI remodeling disrupts this organization in region-specific ways, creating a composite ventricle composed of scar, heterogeneous border tissue, and remodeling remote myocardium.

Infarct core microstructure evolves from myocyte-rich tissue to a collagen-dense scar. Early after MI, the region is characterized by necrosis, edema, and loss of organized contractile machinery, producing low active force and often elevated compliance. As healing proceeds, collagen volume fraction increases substantially, with fibers forming networks whose alignment reflects both biological patterning and mechanical loading during deposition. Cross-link density increases with maturation, and the scar’s mechanical behavior becomes increasingly stiff and less time-dependent. Importantly, scar anisotropy is not guaranteed to align with the original myofiber orientation. Collagen alignment can follow principal strain directions experienced during healing, implying that infarct mechanics reflects the loading history. This creates a mechanistic rationale for why therapies that alter loading early after MI can influence later remodeling: they change the strain field that guides collagen deposition and alignment.

The infarct border zone is a spatial transition with complex microstructure. It typically contains viable myocytes interspersed with fibrosis, inflammatory components, and microvascular changes [47,48,49,50]. Myocyte architecture may be disrupted; fiber orientation can deviate from healthy patterns; and laminar sheet structure may be altered. Border zone collagen may appear as interstitial fibrosis, replacement fibrosis, and perivascular fibrosis, each contributing differently to mechanics. Because viable myocytes persist, border zone tissue exhibits active contraction, but contraction is often reduced and asynchronous due to altered electrophysiology and calcium handling. These features create a distinctive mechanical signature: border zone deformation reflects a combination of passive stiffness heterogeneity, impaired active tension, and strong coupling to neighboring scar and remote tissue [47]. From a modeling standpoint, treating the border zone as a simple interpolation between scar and remote myocardium is attractive but may miss the key physics, because the border zone may have qualitatively different anisotropy and active behavior rather than merely intermediate values [88,89,90,91,92,93,94,95].

Remote myocardium is often assumed to be “healthy,” but post-MI it remodels in a manner that is itself microstructure dependent. Increased wall stress can trigger myocyte hypertrophy, which changes wall thickness and thus the stress distribution. Over time, neurohormonal activation and mechanical strain can promote diffuse interstitial fibrosis. Collagen accumulation increases passive stiffness and impairs diastolic relaxation, while changes in titin isoforms and phosphorylation can shift the low-strain stiffness of cardiomyocytes. Remote remodeling is therefore both structural and molecular, and its mechanics cannot always be represented by a fixed healthy constitutive law. For prediction tasks focused on chronic remodeling, remote tissue properties are often as important as scar properties, because remote stiffness and contractility largely determine diastolic filling and global pump function [96,97].

Quantifying microstructure for modeling is straightforward in controlled experimental settings but challenging in vivo. Histology provides collagen content, fiber orientation, and dispersion, and can be combined with polarized light microscopy or second-harmonic generation imaging to quantify alignment and waviness. Such measurements have enabled mechanistic links between collagen architecture and macroscopic stiffness in animal models and in ex vivo human tissue. In patients, microstructure must typically be inferred from imaging proxies. LGE CMR provides scar distribution and transmurality, but its relationship to stiffness is indirect and depends on collagen architecture and maturation. T1 mapping and extracellular volume estimates can reflect diffuse fibrosis but must be calibrated against collagen content and mechanical properties. Diffusion MRI provides fiber orientation in research contexts, and emerging elastography approaches may provide in vivo stiffness proxies, but interpretation in an anisotropic, actively contracting organ remains nontrivial. These limitations motivate the use of microstructure-informed priors rather than direct microstructure measurement in most patient-specific models, and they highlight why uncertainty must be propagated when microstructure is inferred indirectly [98,99,100].

## 5. Constitutive Models for Post-MI Ventricular Mechanics: Region Specificity and Model-Form Uncertainty

Most ventricular mechanics models combine a passive material law with an active contraction description for viable myocardium [101,102,103,104]. Passive laws are commonly hyperelastic, reflecting the largely elastic behavior observed in quasi-static myocardial testing at physiological time scales, and they encode anisotropy via preferred directions associated with fibers and, in more detailed models, sheets [101,102,103,105,106,107]. Active behavior is often represented via an active stress that adds to the passive stress in the fiber direction or via an active strain formulation that represents contraction as an inelastic deformation. Post-MI modeling must decide how these components vary across infarct, border zone, and remote myocardium and how they evolve over time [35,45,95].

For remote myocardium, transversely isotropic exponential models and orthotropic models have been used widely. These laws can fit biaxial and shear data and can be tuned to reproduce physiological pressure–volume behavior in an FE ventricle [45]. The key practical issue is parameter count. Rich orthotropic laws may represent fiber, sheet, and sheet-normal stiffness, but clinical data rarely constrain all parameters. Consequently, many patient-specific models use a reduced form, sometimes assuming fixed anisotropy ratios and estimating a single stiffness scale. Such simplification can improve identifiability, but it also introduces model discrepancy if the assumed ratios are wrong, particularly in remodeled remote tissue where fibrosis and myocyte changes can alter anisotropy [35,95].

Infarct scar is typically represented as a passive material with higher stiffness than remote myocardium [101,102,103]. The simplest approach is isotropic stiffening, justified by limited in vivo information about scar anisotropy. Yet experimental studies suggest that scar can be anisotropic, with collagen alignment producing direction-dependent stiffness. Representing scar as isotropic may therefore bias stress predictions and influence simulated infarct expansion. The degree to which this matters depends on the context of use. For predicting global volumes, isotropy may be adequate; for predicting rupture risk or stress concentrations at the infarct–border interface, anisotropy may be important. Time dependence is also relevant. Acute infarct tissue may exhibit poroelastic effects due to edema, and subacute scar may exhibit evolving viscoelasticity as matrix matures. Models that treat infarct stiffness as static may therefore misrepresent early-phase mechanics.

Border zone modeling is arguably the most challenging because the region is both passively heterogeneous and actively impaired. Some models represent it as a spatially varying mixture between scar and remote myocardium, with stiffness and activation interpolated based on a distance-to-scar field. This yields smooth parameter maps that are numerically stable. However, histology suggests that border zone fibrosis is patchy and may have anisotropy patterns that are not smoothly varying. Moreover, border zone contractility impairment is not purely a function of distance; it is influenced by microvascular obstruction, inflammation, and electrical remodeling. Thus, simplified border zone representations may fit observed strain but misattribute mechanisms. More detailed approaches represent the border zone as a mixture of viable tissue and fibrosis with separate constituents, but these approaches add parameters and exacerbate identifiability challenges [108,109,110,111].

Active contraction models introduce additional uncertainty. Post-MI, active tension generation is absent in the infarct core and impaired in the border zone. Inverse calibration often estimates a regional contractility scaling factor. Yet contractility interacts with stiffness and loading, so estimates can be non-unique. In addition, active behavior depends on activation timing and electromechanical coupling, which can be altered after MI and by therapies. Many mechanical models prescribe activation timing, which may be acceptable for quasi-static fitting but problematic for predicting systolic dynamics or dyssynchrony. When models include active behavior, it is crucial to state what aspects are estimated from data and what aspects are assumed [46,112,113,114,115,116].

Model-form uncertainty is therefore unavoidable. Different constitutive choices can fit the same data with different parameter values, producing different stress predictions. This is not a weakness of modeling per se; it is a statement about data limitations. For translation, model-form uncertainty should be treated explicitly, for example, by comparing a small set of plausible constitutive families and propagating the resulting prediction differences into uncertainty intervals. A clinically credible model is not one that asserts a single truth, but one that quantifies plausible ranges of stress and remodeling predictions given the available evidence [55,117,118,119,120,121,122,123,124,125,126,127].

Table 2 provides a concise, region-specific view of the constitutive modelling “design space” typically adopted in post-MI ventricular simulations, and crucially, makes explicit how each modelling choice conditions the inverse problem and its dominant failure modes. For remote myocardium, transversely isotropic (or reduced orthotropic) hyperelastic laws coupled to rule-based or diffusion MRI-informed fiber fields and an active stress/strain formulation (often scaled by a contractility factor) are widely used; however, without pressure constraints, passive stiffness and contractility remain strongly confounded, so apparently good kinematic fits can correspond to materially different inferred parameter combinations. Border-zone representations commonly increase complexity via spatially varying parameters or mixture-based formulations to reflect heterogeneity, but this is paired with simplified anisotropy and reduced/prescribed activation timing, creating a recurring non-uniqueness in which scar stiffness and border-zone contractility can trade off to reproduce similar strain patterns. In the infarct scar, stiffened passive laws are often combined with isotropy for practicality. Yet, this assumption can bias predicted stress concentrations and misrepresent interface mechanics, particularly when the modelling objective depends on local stress gradients rather than global volumes. Finally, Table 2 highlights that interface handling (smooth interpolation versus discontinuous partitions) is not a neutral numerical choice: predictions can be sensitive to smoothing length scale and mesh resolution, so discretisation and regularisation should be treated as part of the modelling assumption set when interpreting stress-based biomarkers or remodelling cues.

## 6. Growth and Remodeling Frameworks for Healing and Chronic Remodeling

Growth and remodeling (G&R) models aim to represent how the myocardium’s structure and material properties evolve in response to mechanical and biochemical stimuli. After MI, three remodeling processes are particularly important: scar formation and maturation within the infarct, geometric remodeling of the ventricle including dilation and wall thinning or thickening, and remodeling of the remote myocardium via hypertrophy and diffuse fibrosis. These processes have distinct time scales and are driven by overlapping stimuli. The challenge is to write mathematical laws that are sufficiently mechanistic to generalize yet sufficiently constrained to be identifiable from available data [2,4,5,6,12,34,77].

Constituent-based mixture theories provide a biologically interpretable framework. In these models, tissue is treated as a mixture of constituents such as myocytes and one or more collagen fiber families, each with its own stress response and turnover dynamics [54]. New collagen can be deposited with a specified deposition stretch and preferred orientation, and existing collagen can degrade according to a rate law. Mechanical feedback enters through stress- or strain-dependent synthesis or degradation rates. Such models can represent scar maturation by increasing collagen mass fraction and cross-link maturity, and they can represent fiber reorientation by allowing newly deposited fibers to align with principal strain directions. These models provide a direct link between microstructure and macroscopic behavior and can generate emergent anisotropy. However, they introduce many parameters, including turnover rates and deposition stretches, that are difficult to infer in vivo [2,4,5,6,12,34,77].

Phenomenological growth tensor approaches represent growth as an inelastic deformation that modifies the tissue’s natural configuration. The deformation gradient is decomposed into elastic and growth parts, and a growth law specifies how the growth tensor evolves in response to mechanical stimuli such as stress or strain. Such models are computationally convenient for simulating changes in wall thickness and chamber dilation. They can represent hypertrophy as growth in the fiber direction or as isotropic growth. Yet without explicit constituent interpretation, connecting the growth law to measurable biology can be challenging. For example, a law that increases growth in response to elevated wall stress may reproduce dilation, but it may not specify whether dilation arises from myocyte elongation, collagen degradation, or changes in residual stress [2,4,5,6,12,34,77].

In infarct healing, a key mechanistic question is how infarct stiffness evolves. Experimental studies show that infarct material properties change substantially over time, with early compliance and later stiffening as collagen accumulates and matures. G&R models can represent this by time-dependent scaling of stiffness parameters, by increasing collagen mass fraction in a mixture model, or by coupling stiffness evolution to mechanical stretch experienced during healing. An appealing hypothesis is that collagen alignment follows the mechanical loading pattern, creating an anisotropic scar whose stiffness directions align with principal strains. Such a mechanism offers an explanation for observed variability in scar mechanics and for the influence of mechanical interventions, such as restraint devices or injectable biomaterials, that alter infarct deformation early [2,4,5,6,12,34,77].

In chronic remodeling, global ventricular dilation and remote hypertrophy reflect a balance between loading, contractility, and structural adaptation. Models often incorporate a homeostatic stress hypothesis: the tissue remodels to restore stress or strain to a preferred range. When stress is elevated, hypertrophy increases wall thickness to reduce stress. When stress is reduced, atrophy or thinning may occur. This concept has strong intuitive appeal but is not always sufficient, because remodeling also depends on neurohormonal factors and on the time-varying stiffness heterogeneity created by the scar. Moreover, restoring stress locally may not restore global pump function. Consequently, G&R models must be evaluated not only by whether they restore a homeostatic variable but by whether they reproduce observed clinical trajectories [2,4,5,6,12,34,77].

A major gap is validation. Many remodeling laws can produce qualitatively plausible results, but few are validated against longitudinal datasets that include both geometry and mechanical biomarkers. Without such data, remodeling parameters remain uncertain. This motivates a shift in emphasis: rather than proposing ever more detailed remodeling laws, the field should prioritize studies that jointly estimate remodeling parameters and quantify uncertainty using longitudinal imaging, and that test predictions prospectively or on held-out cohorts. A remodeling law that is modest but identifiable and validated may be more clinically useful than a complex law that is biologically rich but underconstrained [2,4,5,6,12,34,77].

## 7. Multiscale Coupling: Strategies for Bridging Microstructure and Organ Function

Multiscale modeling is attractive in post-MI mechanics because the critical determinants of stiffness and remodeling are microstructural, yet clinical outcomes are organ-level. The challenge is to connect these scales without creating an intractable model. Practical multiscale coupling therefore relies on selective detail: represent microstructure in the aspects that most strongly influence organ-level mechanics, and treat other aspects as uncertainty to be bounded [35,36,45,128,129,130,131,132,133,134,135,136,137,138].

At the microscale, collagen network models capture fiber recruitment, waviness, and cross-linking, all of which shape nonlinear stiffening. Such models can be parameterized from histology and can predict how changes in collagen architecture alter stiffness. When used directly, however, they are too expensive for whole-heart simulation. As a result, microstructure is often encoded indirectly through parameters such as fiber dispersion, collagen volume fraction, and preferred orientation. Structurally based constitutive laws provide a middle ground, allowing parameters to be interpreted in microstructural terms [36].

At the mesoscale, myocardial fiber architecture rotates through the wall, and sheets provide planes of shear. Rule-based fiber assignment is widely used because in vivo measurement is difficult. Yet after MI, fiber architecture can be altered locally, and the infarct can disrupt normal patterns. Diffusion MRI provides direct fiber information in ex vivo studies and in selected in vivo research settings. When diffusion data are unavailable, it is important to propagate uncertainty in fiber architecture, because fiber direction strongly influences predicted stress. A practical approach is to generate an ensemble of plausible fiber fields consistent with anatomy and to quantify how predictions vary across the ensemble [35,36,39,41,101,102,103,105,106,138,139,140,141,142].

At the organ scale, FE models integrate geometry and boundary conditions. For patient-specific models, geometry is often the most reliable input, but boundary conditions and material properties are not. The multiscale perspective suggests that some parameter values should be constrained by microstructure-informed priors. For example, infarct stiffness should be constrained to ranges consistent with scar collagen content and maturity, rather than being allowed to vary arbitrarily to fit kinematics. Similarly, remote stiffness changes implied by diffuse fibrosis biomarkers can constrain priors on passive parameters. This converts personalization into a constrained inference problem, reducing non-uniqueness [35,36,39,41,101,102,103,105,106,138,139,140,141,142].

Reduced-order modeling is another multiscale strategy. Many clinical tasks require rapid computation and repeated simulation for uncertainty quantification (UQ) or therapy comparison. Surrogate models can approximate FE outputs as a function of key parameters, enabling Bayesian calibration or sensitivity analysis. The central requirement is that surrogate models be trained and validated on physically relevant parameter ranges and that they preserve monotonicity and stability constraints. If a surrogate is used to make clinical decisions, its error becomes part of model uncertainty and must be quantified [35,36,39,41,101,102,103,105,106,138,139,140,141,142].

Ultimately, multiscale coupling in post-MI remodeling is less about building a single “complete” model and more about integrating information so that inferences are constrained, uncertainties are explicit, and predictions are relevant to clinical decisions [35,36,39,41,101,102,103,105,106,138,139,140,141,142].

## 8. Imaging-Informed Personalization Pipelines: From Data to a Calibrated Model

Patient-specific modeling begins with geometry and motion, most commonly derived from cine cardiac magnetic resonance (CMR). Segmentation yields ventricular surfaces and wall thickness, and time-resolved volumes provide global kinematics. LGE imaging provides scar distribution, often used to define infarct core and a peri-infarct region. Echocardiography provides an accessible complement, offering high temporal resolution strain estimates and Doppler-derived hemodynamics. CT may be used when CMR is contraindicated or to provide high-resolution anatomy, particularly for the coronary tree and calcification patterns. These data sources are not interchangeable; they provide different information content and have different uncertainties [52,56,57,137,143,144,145,146,147,148,149,150,151,152,153,154,155].

A typical pipeline includes geometry reconstruction, assignment of fiber architecture, selection of constitutive laws, application of boundary conditions, and calibration. Geometry reconstruction is increasingly automated but still a significant source of uncertainty, especially near the base and in the right ventricular insertion regions. Fiber architecture is often assigned using rule-based transmural rotation patterns. When diffusion MRI is available, it can provide fiber directions, but diffusion-derived fibers have their own uncertainties due to resolution and motion artifacts. A practical approach is to treat fiber architecture as an uncertain input and quantify how it influences fitted parameters [52,56,57,137,143,144,145,146,147,148,149,150,151,152,153,154,155].

Strain estimation provides regional deformation constraints. CMR tagging, DENSE, feature tracking, and echocardiographic speckle tracking produce strain estimates with different spatial and temporal fidelity. For inverse calibration, strains should ideally be represented with uncertainty, reflecting tracking error and smoothing assumptions. Pressure loading is often the limiting factor. Many studies use brachial cuff pressure as a proxy for LV pressure, which can be reasonable for end-systolic pressure but less reliable for end-diastolic pressure and for patients with valvular disease or altered arterial compliance. When invasive pressure is available, it dramatically improves identifiability. When it is not, probabilistic pressure priors and hemodynamic models can provide uncertainty bounds rather than single values [154,155,156,157,158,159,160,161,162].

Calibration typically aims to match global volume curves and regional strain patterns. Optimization-based calibration provides point estimates but can converge to local minima and can mask non-uniqueness. Bayesian calibration provides parameter distributions but is computationally expensive. Regardless of approach, identifiability analysis is critical: one should test whether the chosen data set can distinguish the parameters being estimated. If not, parameters should be fixed to plausible priors or the model should be simplified [52,56,57,137,143,144,145,146,147,148,149,150,151,152,153,154,155]. A clinically oriented pipeline must also manage workflow constraints. The time required for segmentation, strain estimation, and model calibration must be compatible with clinical timelines. This motivates model reduction, use of surrogate models, and standardized data processing. In the longer term, the most valuable pipelines may be those that produce not only a best-fit model but also a quantified uncertainty on stress and remodeling predictions, enabling risk-aware clinical interpretation [52,56,57,137,143,144,145,146,147,148,149,150,151,152,153,154,155]. Recent reviews further synthesize the clinical role of multimodality viability assessment and the practical strengths/limitations of speckle-tracking strain for routine deformation measurement, reinforcing the need to carry measurement uncertainty into inverse inference [163,164].

As summarised in Table 3, patient-specific post-MI ventricular mechanics pipelines are fundamentally data-constrained inverse problems in which each measurement modality restricts a different subset of the feasible parameter space, while simultaneously injecting modality-specific uncertainty that must be propagated to avoid overconfident stress and remodelling forecasts. Cine CMR anchors ventricular geometry, volumes, and wall thickness—thereby constraining global kinematics and chamber shape—but offers no direct stiffness information, making segmentation and temporal-resolution errors a primary upstream driver of uncertainty. LGE-CMR provides the scar map and transmurality needed to partition infarcted regions and set heterogeneity priors, yet threshold dependence, timing sensitivity, and partial-volume effects introduce classification uncertainty that can materially alter inferred scar–border contrast. T1/ECV mapping provides a proxy for diffuse fibrosis that can inform priors on remote stiffness and collagen content. Still, its mechanical interpretation is indirect and potentially confounded (e.g., by oedema), so sequence dependence and calibration uncertainty must be carried forward. Regional strain time series from tagging/DENSE/feature tracking (and, longitudinally, echocardiographic strain with Doppler hemodynamics) supply the strongest deformation constraints for inverse fitting. Still, tracking bias, smoothing choices, and inter-observer variability can masquerade as accurate spatial gradients in stiffness or activation if treated deterministically. Finally, LV pressure is often the limiting constraint: when invasive pressure is available, it strongly conditions both passive and active inference, whereas in its absence, timing alignment and measurement drift (or proxy-pressure assumptions) require explicit probabilistic treatment to prevent confounding by stiffness–pressure and stiffness–contractility. Collectively, Table 3 motivates a shift from “best-fit” personalisation to uncertainty-aware inference, in which segmentation, classification, tracking, operator, and loading uncertainties are represented explicitly and propagated through calibration to yield credible predictive intervals aligned to the clinical context of use [35,131,136,137,138,139,140,141,142,143,144,145,146,147,148,149,150].

## 9. Parameter Identifiability in Post-MI Models: What Can Be Learned from Clinical Data?

Identifiability determines whether a patient-specific model can support clinical inference [57,58,59]. In post-MI mechanics, the inverse problem is to infer passive stiffness, anisotropy, and active function in multiple regions from a limited set of observables [22,45]. Even with high-quality geometry and strain data, the inverse problem is commonly ill-posed because different parameter combinations produce similar outputs [56,60,61]. This is particularly true when loading conditions are uncertain, as is typical when LV pressure is not measured [55,62,63].

A foundational confounder is the stiffness–pressure trade-off in diastole [55,62,63]. The diastolic pressure–volume relationship can be matched by increasing stiffness while decreasing assumed pressure, or vice versa [56,62]. If pressure is treated as a fixed input but is uncertain, stiffness estimates inherit that uncertainty [60,62,64]. Similarly, in systole, ejection fraction and regional strain patterns reflect both active tension and passive stiffness [45,165]. A region that shortens less could be less contractile, stiffer, more heavily loaded, or some combination [22,45,109]. Without independent information on loading and contractility, the inverse problem cannot separate these effects uniquely [56,57,61,64].

Regional confounding is especially important in post-MI hearts [22,45,105,106,166]. Scar stiffness influences how much the infarct bulges and how stress is redistributed to the border zone. Border zone contractility influences how much adjacent tissue pulls on the scar [22,45,105,106,109,166]. Many combinations of scar stiffness and border zone contractility can produce similar border zone strain. Thus, calibrating both parameters simultaneously without additional constraints can yield non-unique solutions [57,64]. This is not merely a numerical inconvenience; it undermines the interpretability of parameter maps.

Identifiability analysis should therefore be an explicit component of model development [57,58,59,64]. Structural identifiability can be assessed using simplified models and symbolic methods, while practical identifiability can be assessed via sensitivity analysis, profile likelihood, or Bayesian posterior analysis. In practice, a useful step is to compute how outputs change with parameters and to identify parameter combinations that are poorly informed. If a parameter cannot be constrained, it should be fixed to a prior distribution and reported as such rather than estimated as a point value [57,58,59,60,64].

Several strategies improve identifiability in clinically realistic settings [22,64]. Incorporating even approximate pressure information with uncertainty reduces stiffness–pressure confounding [55,62,63]. Including multi-phase strain time series rather than a single frame improves separation of passive and active contributions [22,45,109]. Using microstructure-informed priors derived from LGE and T1/ECV mapping restricts scar and fibrosis stiffness parameters to plausible ranges [167,168,169]. When available, invasive pressure or catheter-based measurements can substantially improve inference and serve as validation. Finally, reducing model complexity to match data informativeness is often a virtue rather than a limitation [57,59,64,94]. A simpler model that yields identifiable parameters with quantified uncertainty can be more clinically useful than a complex model that produces unstable or non-unique estimates [55,57,59,61].

The broader implication is that patient-specific prediction requires a shift from “fitting” to “inference” [56,60,61]. Fitting is optimized for agreement with observed kinematics; inference is extracting parameter information and making predictions with uncertainty [56,57,170]. The latter is essential for clinical credibility [59,92].

**Figure 3:**
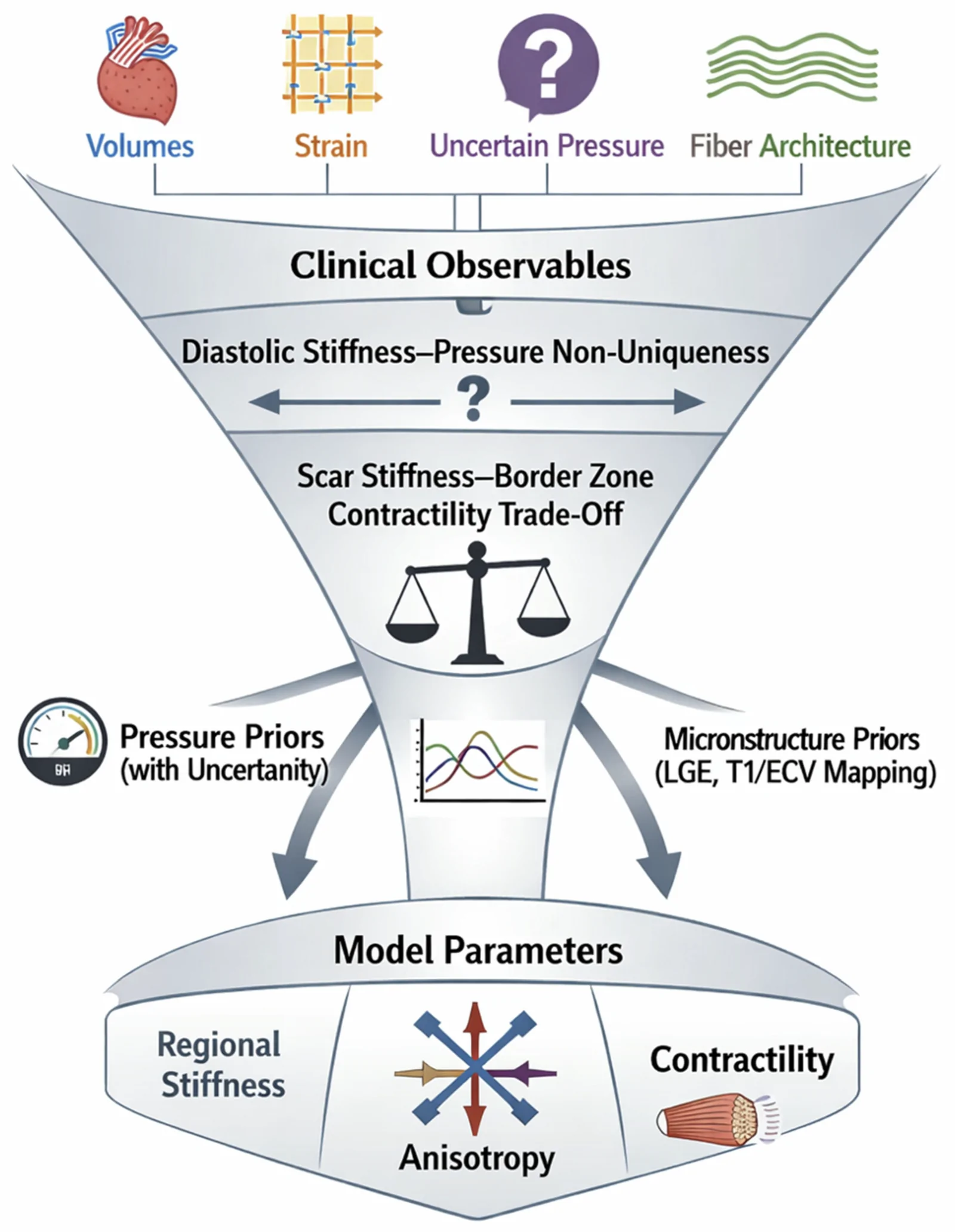
Conceptual funnel of identifiability in post-MI ventricular mechanics, from clinical observables to model parameters. The top of the funnel represents common clinical inputs, including ventricular volumes, limited strain measurements, uncertain pressure data, and poorly constrained fibre architecture. The bottom represents the region-specific parameters to be inferred, such as passive stiffness, anisotropy, and active contractility in the infarct, border zone, and remote myocardium. Key confounders include stiffness–pressure non-uniqueness during diastole and trade-offs between scar stiffness and border-zone contractility. Additional constraints, including pressure priors, multi-phase strain data, and LGE- or T1/ECV-informed fibrosis measures, narrow the feasible parameter space and improve predictive credibility. Abbreviations: LGE, late gadolinium enhancement; ECV, extracellular volume fraction.

As summarized in Figure 3, identifiability in post-MI ventricular mechanics can be interpreted as funnel mapping imperfect clinical observables to a high-dimensional parameter space. In typical workflows, volumes and partial strain measurements are available, while key drivers such as LV pressure (particularly diastolic filling pressure) and patient-specific fiber architecture remain uncertain; consequently, inverse calibration admits multiple parameter sets that fit the data comparably well. The figure highlights two recurring sources of non-uniqueness: the diastolic stiffness–pressure confounder, where passive stiffness can be traded against assumed pressure to match the diastolic pressure–volume relationship, and the scar stiffness–border zone contractility trade-off, where impaired regional shortening can be attributed to altered passive properties, reduced active tension, or both. Importantly, the funnel narrows, and model-based predictions become more decision-credible—when the measurement set is enriched with explicit pressure priors (with uncertainty), time-resolved multi-phase strain information, and microstructure-informed priors derived from LGE and T1/ECV mapping, which collectively constrain the modes of variability responsible for these confounders.

Table 4 summarises why apparently “well-fitting” post-MI ventricular mechanics personalisations can still fail to support reliable clinical inference: with typical imaging-driven observables, multiple parameter combinations can reproduce the same kinematics, so inverse calibration admits non-unique solutions even when residual errors are small. In particular, diastolic stiffness–pressure confounding allows similar pressure–volume behaviour to be matched by trading assumed filling pressure against passive stiffness, producing a wide range of fitted stiffness values with comparable error; a practical mitigation is to treat pressure as uncertain, incorporate filling dynamics where available, and enforce physiologic priors with explicit sensitivity checks. Likewise, scar stiffness–border zone contractility confounding reflects a passive–active trade-off in regional motion that yields non-unique parameter maps; this can be reduced by leveraging multi-time strain information, incorporating viability/perfusion-informed priors, and constraining scar properties by maturation stage. Additional non-identifiability arises from uncertain fibre architecture (strong sensitivity of stress/strain to fibre directions) and boundary condition ambiguity (basal constraints and pericardial effects), motivating uncertainty propagation via fibre ensembles or diffusion MRI when feasible, along with calibration of basal constraints, torsion validation, and simplified pericardial contact representations. Finally, Table 5 highlights that overparameterised constitutive laws amplify optimisation instability and posterior uncertainty, so model reduction, model selection, and microstructure-informed priors are often necessary to align model complexity with the informativeness of routine clinical datasets.

## 10. Uncertainty Quantification and Credibility: Verification, Validation, and Decision Relevance

Uncertainty quantification is a prerequisite for translating post-MI mechanics models into clinical settings because decisions must be made under uncertainty. Uncertainty enters through imaging segmentation, strain estimation, pressure assumptions, fiber architecture, constitutive model choice, and numerical discretization. If these uncertainties are not represented, models may produce stress or remodeling predictions that appear precise but are in fact fragile [170,171,172,173,174,175,176,177].

A practical UQ workflow begins with verification. Numerical verification includes mesh convergence and solver robustness checks, ensuring that predicted stresses and strains are not artifacts of discretization. Verification is often neglected in patient-specific contexts due to time constraints, yet it is particularly important near the infarct–border interface where stress gradients are steep. Next is validation. Validation requires comparison to independent observables, not those used for calibration. For example, if a model is calibrated to volumes and strains, it can be validated against torsion, wall thickening, or hemodynamic measures not used in the fit. In animal models, validation can include direct measurement of regional strains via implanted markers or measurement of material properties ex vivo. In patients, validation is more constrained but can still be performed using longitudinal follow-up and cross-modality comparisons [170,171,172,173,174,175,176,177].

Parameter uncertainty can be quantified via Bayesian inference or via ensemble approaches. Bayesian inference yields posterior distributions but is computationally expensive for FE models. Ensemble approaches, such as sampling plausible parameter sets that fit within an error tolerance, can approximate uncertainty. Model-form uncertainty is often larger than parameter uncertainty and can be assessed by comparing a small number of plausible constitutive families or boundary condition models. If different model forms produce materially different stress predictions, this variation should be included in uncertainty intervals [170,171,172,173,174,175,176,177].

For clinical decision support, uncertainty must be connected to decision thresholds. If a model is used to classify risk of adverse remodeling, the output might be a probability that end-systolic volume will exceed a threshold at six months. Such probabilities require uncertainty propagation. Similarly, for therapy planning, uncertainty should be propagated through counterfactual simulations to quantify how robust a predicted benefit is to parameter uncertainty. The goal is not to eliminate uncertainty, which is impossible, but to represent it in a way that supports robust decisions [170,171,172,173,174,175,176,177].

Credibility frameworks developed for computational models in medicine emphasize context of use, verification, validation, and uncertainty. For post-MI mechanics, a context of use might be early prediction of adverse remodeling, selection of patients for ventricular restraint, or planning of surgical restoration. Each context implies required accuracy for specific quantities, such as volume change or regional stress. Aligning model development with a context of use focuses measurement priorities and validation design. Without this alignment, models risk being impressive but clinically irrelevant [170,171,172,173,174,175,176,177].

## 11. What to Measure Next: Building Datasets That Make Prediction Possible

A recurring theme in post-MI mechanics is that models are ahead of data. Many modeling frameworks can represent plausible remodeling, but few datasets can constrain and validate them. A pragmatic measurement agenda should therefore prioritize combinations of measurements that increase identifiability and enable validation, rather than simply collecting more of the same data.

Paired pressure–volume–strain datasets are a high priority. Pressure is the missing variable in many patient-specific studies, and its uncertainty drives stiffness uncertainty. When invasive pressure is not feasible, pressure should be estimated using physiologically grounded methods with uncertainty bounds, and the uncertainty should be carried into the inference. Strain should be measured with methods that provide reproducible regional patterns and should include uncertainty estimates. Through-wall strain components remain difficult in clinical imaging but are valuable because they constrain anisotropy and laminar mechanics. Even partial improvements in through-wall information could materially improve identifiability.

Imaging-to-mechanics calibration studies are also essential. LGE and T1/ECV mapping are widely available, but their mechanical meaning is not fixed. Studies that link these imaging biomarkers to ex vivo stiffness and anisotropy, across maturation stages, would provide priors that constrain scar and remote stiffness. Such calibration should explicitly quantify variability and measurement dependence on scanner, sequence, and timing. Similar calibration is needed for emerging elastography approaches, which provide stiffness proxies that are frequency dependent and require model-based interpretation in anisotropic tissue.

Longitudinal imaging is crucial for remodeling prediction. A model calibrated to a single time point can fit a snapshot but cannot be tested on its ability to predict change. Longitudinal follow-up at clinically meaningful intervals, such as baseline soon after MI, one month, three months, and six months, can constrain remodeling parameters and enable out-of-sample validation. Even two time points provide a stringent test: can the model predict the direction and magnitude of volume change? Longitudinal data are also needed to validate the timing of infarct stiffening and remote fibrosis progression.

Validation endpoints should be selected to match clinical decisions. If the decision is risk of adverse remodeling, endpoints may include end-systolic volume increase, decline in ejection fraction, or onset of heart failure hospitalization. If the decision is surgical or device planning, endpoints may include regional wall stress or predicted improvement under intervention. Standardizing endpoints across studies would enable comparability and meta-analysis.

Finally, the field should embrace model reduction guided by data. The most clinically useful model may be the simplest one that is identifiable and predictive for a given context. Reduced models can support faster inference and UQ, making them more feasible in clinical workflows. The emphasis should shift from demonstrating the capacity to simulate post-MI mechanics to demonstrating the capacity to predict clinically relevant outcomes with quantified uncertainty.

### Illustrative Clinical Use Cases for Decision-Relevant Modeling

Use case 1: Early post-MI risk stratification

As a hypothetical example, consider a patient assessed within 1–2 weeks after MI using cine CMR for ventricular volumes, LGE for scar geometry and transmurality, and feature tracking, DENSE, or tagging for regional strain. A patient-specific mechanics model could integrate these data to estimate the probability of adverse remodeling, such as a clinically meaningful increase in end-systolic volume at 6 months.

This model output would not replace established predictors such as LVEF, infarct size, microvascular obstruction, global or regional strain, and CMR-derived remodeling markers. Rather, it could add complementary mechanistic information by estimating regional stiffness, contractile impairment, wall stress, and the combined influence of loading conditions and tissue properties. A high predicted risk, reported with uncertainty, could potentially support more intensive follow-up, repeat imaging, and optimization of guideline-directed therapy. This remains an illustrative future application and is not presented as a currently validated clinical workflow.

Use case 2: Patient selection for mechanically targeted interventions

A second hypothetical application concerns patient selection and trial enrichment for interventions that modify ventricular mechanics, such as infarct-reinforcement therapies, ventricular-restraint devices, or surgical ventricular restoration. Patient-specific models could simulate counterfactual intervention scenarios by varying infarct stiffness, geometry, or loading conditions and estimating the probability of reducing wall stress or adverse remodeling.

Unlike standard predictors, which primarily describe existing injury and ventricular function, these simulations could provide treatment-specific estimates of likely mechanical response. They may therefore complement LVEF, infarct size, microvascular obstruction, strain, and other CMR markers when identifying patients most likely to benefit. In research settings, such predictions could support candidate selection, reduce the inclusion of patients with a low probability of response, and enrich trials for participants in whom a detectable mechanistic benefit is expected. These applications remain exploratory and would require prospective validation before clinical implementation.

## 12. Translational Outlook: From Mechanistic Models to Clinical Tools

Clinical translation requires that post-MI mechanics models provide information that changes decisions or improves outcomes. One promising near-term application is risk stratification. If early post-MI imaging and hemodynamic data can be integrated to predict the probability of adverse remodeling, clinicians could intensify therapy or surveillance for high-risk patients. The added value of a mechanics model would be to provide mechanistically grounded risk indicators such as elevated border zone stress or predicted infarct expansion tendency, which may capture risk not fully explained by infarct size alone [1,23,24,25,178,179,180].

A second application is therapy planning for interventions that directly alter mechanics. Examples include devices that restrain the ventricle, biomaterial injections intended to stiffen the infarct, and surgical ventricular restoration. These interventions change the mechanical environment and thus potentially the remodeling trajectory. Mechanistic models can simulate counterfactual scenarios to estimate benefit and to identify which patients are most likely to respond. However, counterfactual prediction magnifies uncertainty because it extrapolates beyond observed data. Therefore, UQ is not optional in therapy planning; it is central.

Clinical adoption also depends on workflow feasibility and interpretability. Automated segmentation and strain estimation, standardized pressure estimation, and robust model calibration are required. Surrogate models may enable rapid computation, but they must be anchored to physics and validated. Interpretability requires that model outputs be presented in clinically meaningful terms, such as predicted volume trajectory with uncertainty, rather than raw parameter values. It also requires careful communication: a model output is a probabilistic forecast, not a deterministic truth.

From a research perspective, the most valuable progress will come from prospective studies that integrate patient-specific modeling into longitudinal follow-up, test predictions on future outcomes, and refine models based on observed discrepancies. Such studies will also clarify which model components truly matter for clinical decisions and which add complexity without improving prediction [1,19,24].

**Figure 4:**
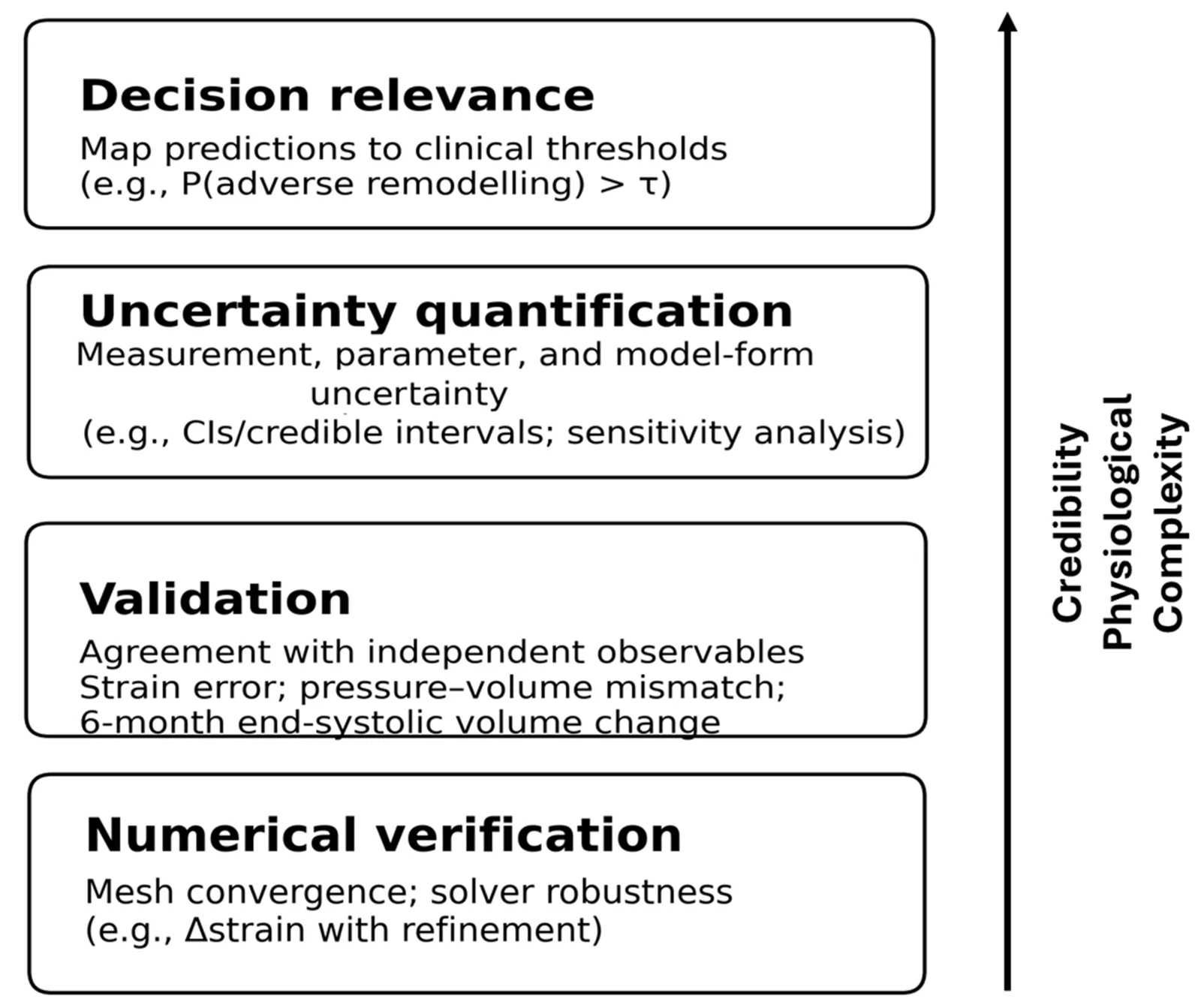
Credibility ladder for patient-specific post-MI ventricular mechanics models aligned to context of use. The ladder progresses from numerical verification and validation against independent observations to uncertainty quantification and clinical decision relevance. It emphasises that greater physiological complexity does not necessarily improve credibility unless each stage is supported by traceable, context-specific evidence and uncertainty-aware predictions [83,84,85,120].

Figure 4 summarises a context-of-use–aligned credibility ladder for patient-specific post-MI mechanics, making explicit that model “realism” is not a substitute for evidence. The ladder begins with numerical verification (mesh convergence and solver robustness), because unstable discretisation can masquerade as physiology; it then requires validation against independent observables and longitudinal outcomes using transparent metrics such as regional strain error, pressure–volume mismatch, and prediction of six-month end-systolic volume change. Crucially, the framework elevates uncertainty quantification, partitioning measurement, parameter, and model-form uncertainty before any claims of clinical utility are made, and it ends with decision relevance, where predictions are translated into clinically meaningful probabilities relative to thresholds rather than point estimates. In combination, these rungs provide a practical audit trail for “decision-grade” modelling and clarify why increasing physiological complexity (e.g., adding microstructure or coupled electromechanics) can still yield low-credibility predictions when verification, validation, and uncertainty controls are weak or absent [83,84,85,120].

## 13. Conclusions

Post-MI remodeling is a multiscale mechanical process driven by evolving microstructure, regional heterogeneity, and global loading. Constitutive and growth-and-remodeling models have reached a level of sophistication that enables patient-specific simulation, yet clinically credible prediction is constrained by limited in vivo observability, parameter non-identifiability, and incomplete uncertainty quantification. The path to translation is therefore clear in principle: align model complexity with data informativeness, incorporate microstructure-informed priors, treat pressure and fiber architecture as uncertain inputs, perform explicit identifiability analysis, and validate predictions against longitudinal, decision-relevant endpoints. Advances in imaging biomarkers, elastography, and data assimilation will help, but the most immediate gains are likely to come from better-designed datasets and from inference practices that quantify uncertainty transparently. In this sense, the next era of post-MI modeling will be defined less by what can be simulated and more by what can be learned, validated, and trusted for clinical decisions.

Three actionable priorities emerge. First, the field needs paired imaging–loading datasets (including pressure information or well-bounded pressure priors) collected with longitudinal endpoints to break stiffness–pressure and passive–active confounders. Second, we need standardized uncertainty reporting for segmentation and strain estimation (including inter-observer variability and method-dependent bias) so that UQ reflects real clinical measurement error rather than idealized noise. Third, progress will accelerate through shared benchmarks and pre-registered validation protocols that test context-of-use predictions (e.g., 6-month remodeling risk) rather than retrospective “best fits,” enabling transparent comparison of model classes and inference pipelines.

The evidence reviewed here indicates that the translational value of microstructure-informed modelling depends less on model complexity alone than on identifiable parameterisation, uncertainty-aware calibration, and validation against clinically relevant endpoints.

## 14. Limitations

This article was conducted as a narrative review and perspective rather than a systematic review. Consequently, the search and study-selection process did not involve formal duplicate screening, prospective recording of all identified and excluded publications, structured resolution of reviewer disagreements, or PRISMA-based study selection. The approximate search and selection counts presented in Figure 2 were reconstructed retrospectively and are intended to improve transparency rather than imply systematic-review methodology.

## Figures and Tables

**Table 1:** Mechanical correlates of the biological phases of post-MI remodeling. The table summarizes characteristic mechanical changes in the infarct core, border zone, and remote myocardium across time. The descriptors reflect trends observed across experimental and clinical studies; timing and magnitude vary by infarct size, reperfusion, and comorbidities. The intent is to guide region- and time-appropriate modeling choices.

Phase (Approx.)	Infarct Core Mechanics	Border Zone Mechanics	Remote Myocardium Mechanics	Dominant Microstructural Drivers
Acute (hours–days)	Loss of active tension; high compliance; high strain; risk of expansion and rupture	High stress gradients; impaired activation; edema-dependent stiffness variability	Load redistribution; elevated stress; early diastolic changes possible	Myocyte necrosis; edema and hemorrhage; early matrix degradation; inflammatory turnover
Proliferative (days–weeks)	Progressive stiffening and strengthening; decreasing expansion tendency	Mixed viable myocytes and fibrosis; heterogeneous anisotropy; high mechanobiological activity	Hypertrophy initiation; onset of diffuse fibrosis in susceptible hearts	Myofibroblast activation; collagen deposition and alignment; early cross-linking; altered titin and cytoskeleton
Chronic (weeks–months)	Stiffer, stronger scar; reduced dissipation; stabilized anisotropy pattern	Patchy fibrosis; altered fiber architecture; arrhythmogenic mechanical substrate	Hypertrophy and diffuse fibrosis increase stiffness; dilation may continue	Collagen maturation and cross-linking; fiber reorientation; myocyte hypertrophy; sustained collagen turnover

**Table 2:** Representative constitutive modeling choices for infarct, border zone, and remote myocardium in post-MI ventricular simulations. The table highlights typical assumptions and their practical implications for parameter inference. The intent is not to prescribe a single model but to align model complexity with data informativeness and context of use.

Region	Common Passive Law	Anisotropy Representation	Active Behavior	Primary Inference Risk
Remote myocardium	Transversely isotropic or reduced orthotropic hyperelastic	Fiber directions from rule-based assignment or diffusion MRI when available	Active stress/strain with length dependence; often scaled by contractility factor	Passive stiffness and contractility can be confounded without pressure constraints
Border zone	Spatially varying hyperelastic parameters or mixture-based formulation	Heterogeneous anisotropy; often simplified to transversely isotropic	Reduced and heterogeneous activation; timing often prescribed	Scar stiffness–border contractility trade-offs produce non-unique fits
Infarct scar	Stiffened hyperelastic; time-dependent scaling for healing phase in some studies	Often isotropic; sometimes transversely isotropic aligned with principal strain or assumed fiber field	None	Isotropic assumption can bias stress concentrations and interface mechanics
Interface and transition	Smooth interpolation fields or discontinuous partitions with constraints	Gradients or discontinuities in fiber/stiffness	Transition in activation magnitude	Predictions sensitive to smoothing length scale and mesh resolution

**Table 3:** Clinical and experimental data sources used to personalize post-MI ventricular mechanics models, and the dominant uncertainty each introduces. The table emphasizes how each measurement constrains the mechanics problem and where uncertainty propagation is essential for credible prediction [52,56,57,137,143,144,145,146,147,148,149,150,151,152,153,154,155].

Data source	Contribution	Mechanics Constraint	Key Limitation	Dominant Uncertainty to Propagate
CMR cine	Geometry, volumes, wall thickness	Constrains global kinematics and chamber shape	No direct stiffness information	Segmentation error; temporal resolution
CMR LGE	Scar map and transmurality	Defines infarct partition; guides heterogeneity priors	Threshold dependence; timing sensitivity	Classification uncertainty and partial volume
CMR T1/ECV	Diffuse fibrosis proxy	Prior on remote stiffness and collagen content	Indirect relation to stiffness; confounded by edema	Sequence dependence; calibration uncertainty
CMR tagging/DENSE/feature tracking	Regional strain time series	Constrains deformation field for inverse fitting	Noise and tracking bias; limited through-wall detail	Tracking error and smoothing assumptions
Echocardiographic strain + Doppler	Regional function and hemodynamics	Constrains timing and filling; accessible longitudinally	Operator dependence; acoustic window limits	Inter-observer variability and signal quality
Invasive LV pressure (subset)	True loading	Strongly constrains passive and active inference	Rare in routine follow-up	Timing alignment; measurement drift
Elastography (emerging)	Stiffness proxy	Potential direct constraint on regional stiffness	Frequency dependence; anisotropy interpretation	Modeling assumptions and motion artifacts

**Table 4:** Common identifiability pitfalls in post-MI ventricular mechanics personalization and practical remedies that can be implemented with typical clinical datasets. The remedies focus on measurement design, prior constraints, and model reduction.

Pitfall	Mechanistic Cause	Observed Symptom	Practical Remedy
Diastolic stiffness–pressure confounding	Similar PV behavior can be reproduced by different stiffness–pressure pairs	Wide range of fitted stiffness values with similar error	Treat pressure as uncertain; include filling dynamics; use physiologic priors and sensitivity tests
Scar stiffness–border contractility confounding	Passive and active contributions trade off in regional motion	Non-unique regional parameter maps	Use multi-time strain; incorporate viability/perfusion priors; constrain scar properties by maturation stage
Uncertain fiber architecture	Stress and strain depend strongly on fiber directions	Parameters change markedly under different fiber fields	Propagate fiber uncertainty; use diffusion MRI when feasible; ensemble testing
Boundary condition ambiguity	Basal constraints and pericardial effects alter deformation	Good fit but unrealistic twist or basal motion	Calibrate basal constraints; validate torsion; include simplified pericardial contact models
Overparameterized constitutive law	Too many degrees of freedom relative to data	Optimization instability; large posterior uncertainty	Reduce parameter count; perform model selection; constrain with microstructure priors

**Table 5:** Minimum practical data packages for identifiability (decision-grade inference) in patient-specific post-MI ventricular mechanics. The table operationalizes identifiability as a function of measurement ability to separate dominant confounders (notably stiffness–pressure and passive–active trade-offs), and links each tier to defensible decision outputs.

Data Package	Typical Measurements	Mechanics Constraint	Key Limitation	Decision-Grade Outputs Most Defensible
Package A: Routine, non-invasive	Cine CMR (geometry and LV volume curves); LGE-CMR (scar localization/transmurality); brachial blood pressure (bounded proxy for systolic loading); multi-phase regional strain (feature tracking or speckle-tracking echocardiography, STE)	Constrains global kinematics and relative regional dysfunction; provides scar geometry priors; supports coarse region-wise differences (infarct vs. remote)	Passive stiffness–pressure confounding (no LV pressure/filling pressure); passive–active trade-off (contractility vs. stiffness); boundary-condition uncertainty; strain method bias/variability	Qualitative or probabilistic risk ranking with wide uncertainty; relative indicators of remodeling risk rather than absolute stress-based thresholds
Package B: Enhanced, still non-invasive	Package A plus T1 mapping/ECV (diffuse fibrosis prior); higher-fidelity strain (DENSE/tagging where feasible); Doppler/echo hemodynamics to bound diastolic filling pressures	Narrower bounds on passive stiffness using tissue characterization priors; improved regional calibration; reduced posterior uncertainty; better separation of infarct/border/remote behavior	Residual uncertainty in absolute LV pressure and boundary conditions; remaining passive–active confounding if activation is weakly constrained; inter-method variability in strain and mapping	More credible probabilistic forecasts of remodeling trajectory; improved patient selection for escalation/surveillance; preliminary stress-based metrics with uncertainty bands (not point estimates)
Package C: Reference standard for parameter separation	Packages A/B plus invasive LV pressure waveform (or well-validated pressure estimation); ideally paired pressure–volume data synchronized with strain	Substantially improved separation of passive stiffness vs. loading and passive vs. active contributions; stronger identifiability of region-wise stiffness/contractility scaling; more credible stress/strain field inference	Model-form uncertainty (constitutive and activation choices); spatial heterogeneity remains under-determined if data are sparse; numerical/interface assumptions can affect stress gradients	Decision-relevant stress-based metrics with quantified uncertainty; counterfactual therapy simulations (e.g., infarct reinforcement) with defensible credibility claims; validation against longitudinal endpoints

Abbreviations: CMR, cardiac magnetic resonance; LGE, late gadolinium enhancement; LV, left ventricle; STE, speckle-tracking echocardiography; DENSE, displacement encoding with stimulated echoes; ECV, extracellular volume fraction.

## Data Availability

The original contributions presented in the study are included in the article. Further inquiries can be directed to the corresponding authors.
